# 

**Published:** 1884-08

**Authors:** 


### CONTENTS.

ORIGINAL COMMUNICATIONS: PAGE.

I. The Effect of Noises upon Certain Forms of Deafness. By George Hawley, M D., Chicago ... 97

II. Cellodin as an Embedding Mass. By Boerne Betterman, M. D., Chicago ... 103

III. Clinical and Pathological Reports of Cases of Insanity. By S. V. Clevenger, M. D., Chicago ... 105

IV. A Synopsis of the Medical Botany of Illinois. By I. M. G. Carter, M. A., M. D., PH. B. ... 115

EDITORIAL:

Hebra's Continuous Tepid-Water Bath ... 122
The Cholera Abroad ... 124
Chicago and the Cholera ... 126

BOOK REVIEWS: PAGE.

Practical Manual of Obstetrics ... 133
Handbook of Vertebrate Dissection. Part III. ... 135

SELECTIONS:

Report of Illinois State Board Meeting ... 136
Circular from the Berlin Polyclinic ... 155
Report American Medical Association ... 156
Gun-Shot Wounds of the Small Intestines. By Charles T. Parkes, M. D., Professor of Anatomy in Rush Medical College, Chicago, Ill. ... 161

BOOKS AND PAMPHLETS RECEIVED ... 191

